# Primary Leiomyosarcoma of the Gallbladder

**DOI:** 10.1155/1990/79294

**Published:** 1990

**Authors:** Costas Fotiadis, Alexandros Gugulakis, Lydia Nakopoulou, Michael Sechas

**Affiliations:** 1 2nd Dept. of Propedeutic Surgery Athens University School of Medicine Laikon Hospital Athens Greece; 2 14-16 Ravine Street Athens 115-21 Greece

## Abstract

The case of a 64 year old female who was known to have gallstones is presented. She was admitted to the Hospital following an attack of acute cholecystitis. Ten days after vigorous conservative treatment cholecystectomy was performed. The histological examination showed the presence of the gallbladder leiomyosarcoma. Primary sarcomas of the gallbladder are rare, leiomyosarcoma being the most infrequent type, their preoperative diagnosis almost impossible and their prognosis poor.


*HPB Surgery*, 1990, Vol. 2, pp. 211–214
Reprints available directly from the publisher
Photocopying permitted by license only

© 1990 Harwood Academic Publishers GmbH
Printed in the United Kingdom

### PRIMARY LEIOMYOSARCOMA OF THE GALLBLADDER
### Case report and review of the literature

COSTAS FOTIADIS, ALEXANDROS GUGULAKIS, LYDIA NAKOPOULOU, and MICHAEL SECHAS

*2nd Dept. of Propedeutic Surgery, Athens University School of Medicine, Laikon Hospital, Athens, Greece.*

*(Received 29 March 1989; in final form 5 June 1989)*

The case of a 64 year old female who was known to have gallstones is presented. She was admitted to the Hospital following an attack of acute cholecystitis. Ten days after vigorous conservative treatment cholecystectomy was performed. The histological examination showed the presence of the gallbladder leiomyosarcoma. Primary sarcomas of the gallbladder are rare, leiomyosarcoma being the most infrequent type, their preoperative diagnosis almost impossible and their prognosis poor.

KEY WORDS: Primary sarcomas of gallbladder, leiomyosarcoma, gallbladder empyema

### INTRODUCTION

Primary leiomyosarcoma is the most infrequent type of primary gallbladder carcinomas. It usually presents as cholelithiasis in female patients in the sixth decade of their life.

Accurate diagnosis is established only with histological examination after cholecystectomy.

Like the other types of gallbladder carcinoma, primary leiomyosarcoma carries a poor prognosis[1].

We present our experience with a patient who underwent cholecystectomy, after developing gallbladder empyema. The presence of leiomyosarcoma was shown by histological examination.

### CASE

A 64 year old female was admitted to the Hospital with symptoms and signs of acute cholecystitis. There was nothing special in her family history and she did not report any previous illnesses.

She is a house-wife and smoked 10 cigarettes a day for the last 35 years. No alcohol consumption was reported.

Two months prior to admission she reported a similar afebrile attack of right upper quadrant (R.U.Q.) pain radiating to the back with nausea, for which she was

Reprint requests to: C. Fotiadis M.D. 14–16 Ravine Street, 115–21 Athens.

hospitalized. At this time a liver/biliary tree ultrasound (U/S) was performed and the presence of gallstones and sludge was confirmed. The patient refused surgery (Figure 1).

For the past 48 hours she complained of R.U.Q. pain radiating to the back accompained by nausea, vomiting (containing initially undigested and later bile stained food) and fever reaching 39°C. On clinical examination she had R.U.Q. tenderness, a positive Murphy's sign and a palpable gallbladder. Chest and abdominal X-rays were within normal limits. The laboratory findings were as follows: Haemoglobin 36%, W.B.C. 15,800/mm3, F.B.S. 108mg/dl, B.U.N. 56mg/dl, Na 138 meq/L,K 4,1 meq/L, bilirubin 0.5mg/dl, Alkaline phosphatase 731.U./L, d Gt 14U/L, prothrombin time 13/12 sec.

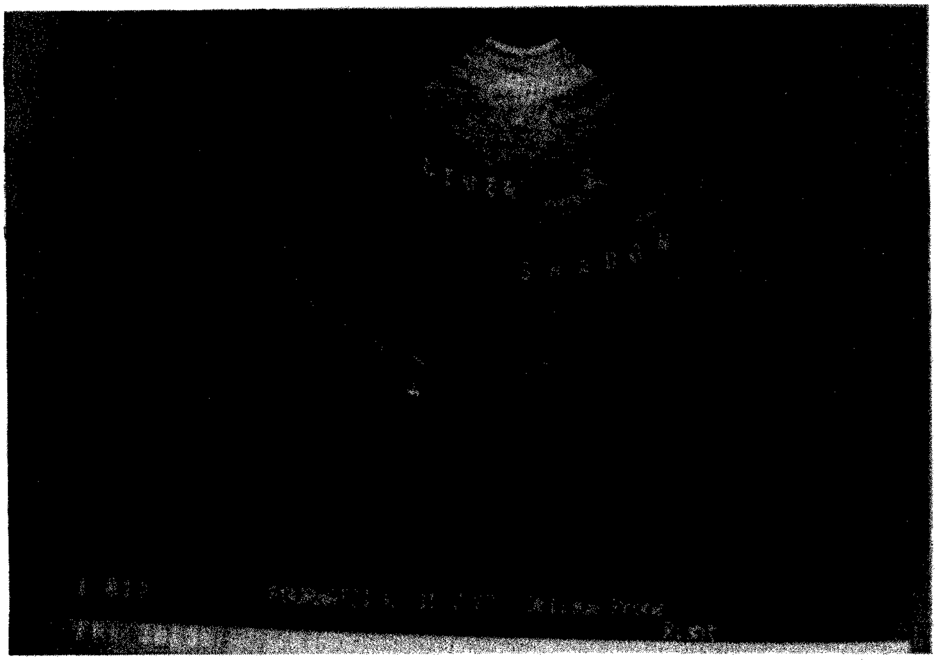

**Figure 1** Gallbladder Ultrasound: Cholelithiasis

The patient was treated with parenteral fluids and electrolyte replacement, broad spectrum antibiotics, spasmolytic agents and analgesics. Forty-eight hours later her condition improved but the gallbladder was still palpable and tender necessitating cholecystectomy on the tenth day after admission. Operative findings were: 1. diffuse inflammatory reaction affecting both the gallbladder and the adjacent liver 2. firm adhesions between gallbladder and the adjacent organs 3. irregular thick and creased gallbladder mucosa and a soft cast filling the gallbladder 4. diverticula-like dilatation just below Hartmann's pouch and 5. a single stone of the mixed type (1.5 by 2.5 cm) stuck into the cystic duct.

The excised specimen consisting of several pieces of the gallbladder was sent for histological examination. The overall dimensions were 7 by 5 by 4 cm and its colour was whitish and of in elastic consistency. The above mentioned cast had a maximal diameter of 5 cm and its colour was yellow-white and it was friable.

Histology showed the presence of leiomyosarcoma consisting of fusiform malignant neoplastic cells with marked atypia, multiformity and many mitosis. The neoplastic cells infiltrated all layers of the gallbladder wall and the surrounding adipose tissue. There were also extensive necrotic areas and haemorrhagic infiltrations while the cast showed a similar picture with scant neoplastic cells in it.

Immunocytochemistry was also performed (peroxidase-anti- peroxidase PAP, Steinberger 1979[2]) on paraffin sections with polyclonal and monoclonal antibodies against desmin and vimentin respectively. Immunopositive staining was strong for both markers in the cytoplasm of many neoplastic cells. (Figure 2)

On the eighth postoperative day, following an uneventful course the patient left the Hospital having refused to be treated with the recommended chemotherapy. She died six month later.

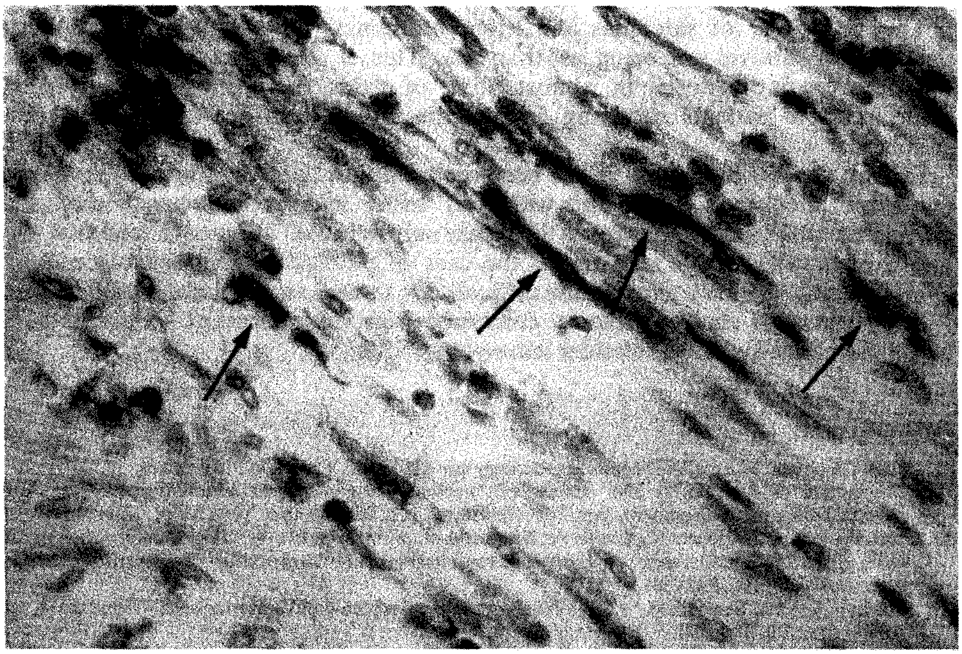

**Figure 2** Gallbladder Leiomyosarcoma: Tumor cells with positive staining for desmin (arrows) (PAPX600)

### DISCUSSION

By 1984, 105 cases of primary sarcomas of the gallbladder have been reported, with primary leiomyosarcomas accounting for 7% of them[3].

These tumors are more common in women in the sixth decade of their life and usually coexist with cholelithiasis (82%)[4,5]..

The main[3] (if not the only) symptom is R.U.Q. pain radiating to the back or to the right lower quadrant, resembling cholecystitis. The nonspecific clinical picture combined with gallbladder stones on the ultrasound leads to the false preoperative diagnosis of cholelithiasis while the true diagnosis is seen only by histology.

There is only one report in the literature in which the presence of some kind of neoplasm was postulated preoperatively. However the exact diagnosis was made again with histological examination[3].

The patient presented here, possibly the ninth in the literature, was diagnosed as an empyema complicating cholelithiasis. The presence of malignancy was suspected during the operation and preliminary confirmed by the frozen section histology when malignancy could not be ruled out. The final diagnosis was based on histology; the differential diagnosis included all histological types of fusiform sarcomas, i.e. fibrosarcoma, neurosarcoma and leiomyosarcoma. The diagnosis of gallbladder leiomyosarcoma was, as previously mentioned, based upon the highly positive immunostaining to desmin and vimentin which are pathognomonic for smooth muscle cells.

The patient succumbed six months later having refused chemotherapy.

The prognosis of sarcomas and leiomyosarcomas of the gallbladder is dismal, the five year survival rate being less than 5%. This is due to the fact that at the time of operation or diagnosis in almost 75% of cases there is liver involvement[1], while in those patients surviving 5 years the tumor does not spread beyond the gallbladder wall.

During the last five years[6] it has been reported that chemotherapy after surgical excision of the tumor in patients with soft tissue leiomyosarcomas prolongs the disease free interval and improves the 5 year survival rate. It is postulated that these improved results may be seen also in the case of gallbladder leiomyosarcomas, but substantiating this may prove quite difficult due to the low incidence of the disease.

At the present cholecystectomy combined with chemotherapy seems to be the treatment of choice for gallbladder leiomyosarcomas.

### *References*

1. Paul Calabresi, M.D. (1985) Medical Oncology pp: 859, 1197, 1206–1207 McMillan.
2. Steinberger L.A., (1979) The unlabelled antibody enzyme peroxidase-antiperoxidase (PAP) method Immunocytochemistry. pp: 104–169 Edited L.A. Steinberger, New York, John Wiley.
3. Newmark H. III, Kliewer K., Curtis A., Denbesten L. and Enenstein W. (1986) Primary Leiomyosarcoma of the Gallbladder seen on computed tomography and Ultrasound. *The American Journal of Gastrenterology*: **81**, 202–204.
4. Yasuma T. and Yanaka M. (1971) Primary sarcoma of the gallbladder-reported three cases. *Acta Pathol.* Jap.: **21**, 285–304.
5. Thorson M. Kristin, Quiroz F., Lawson T.L., Smith D.F., Foley W.D. and Stewart E.T. (1984) Primary Biliary Carcinoma: CT evaluation.
6. Rosenberg S.A., Tepper J., Glutstein E., Costa J., Young R., Baker A., Brennan M.F., Demoss E.V., Seip Claudia R.N., Sindelar W.F., Sugarbaker P. and Wesley R. (1983) Prospective randomized evaluation of adjuvant Chemotherapy in adults with soft tissue sarcoma of the extremities. *Cancer*: **52**, 424–434.

*(Accepted by S. Bengmark 5 June 1989)*

